# The sunflower cataract in Wilson’s disease: pathognomonic sign or rare finding?

**DOI:** 10.1007/s13760-015-0566-1

**Published:** 2015-11-17

**Authors:** Ewa Langwińska-Wośko, Tomasz Litwin, Karolina Dzieżyc, Anna Członkowska

**Affiliations:** 1 Second Department of Neurology, Institute of Psychiatry and Neurology, Sobieskiego 9, 02 957 Warsaw, Poland; 2 Department of Ophthalmology, Medical University of Warsaw, Warsaw, Poland; 3 Department of Experimental and Clinical Pharmacology, Medical University of Warsaw, Warsaw, Poland

**Keywords:** Wilson’s disease, Kayser–Fleischer ring, Sunflower cataract, Lens

## Abstract

The presence of Kayser–Fleischer ring in patients with Wilson’s disease (WD) is well documented and included in diagnostic algorithms; however, data about the occurrence of the second postulated ophthalmological sign of WD, sunflower cataract (SC), are limited and even conflicting. The aim of our study was to verify the occurrence of SC in WD. From January 2010 to May 2015, 81 consecutive, newly diagnosed WD patients underwent detailed ophthalmological examinations, including slit lamp examination with special attention to lens transparency, to verify the presence of SC in WD-naive patients. SC was detected in only one (1.2 %) of the examined WD patients, did not impact visual acuity; moreover, completely disappeared following a year of treatment for WD. SC may be a very rare and reversible ophthalmological manifestation of WD that is observed seldom and only at the time of WD diagnosis. We postulate that a finding of SC in WD patients is an interesting finding that may occur in the course of WD, but it is not a pathognomonic sign of WD.

## Introduction

**Fig. 1:**
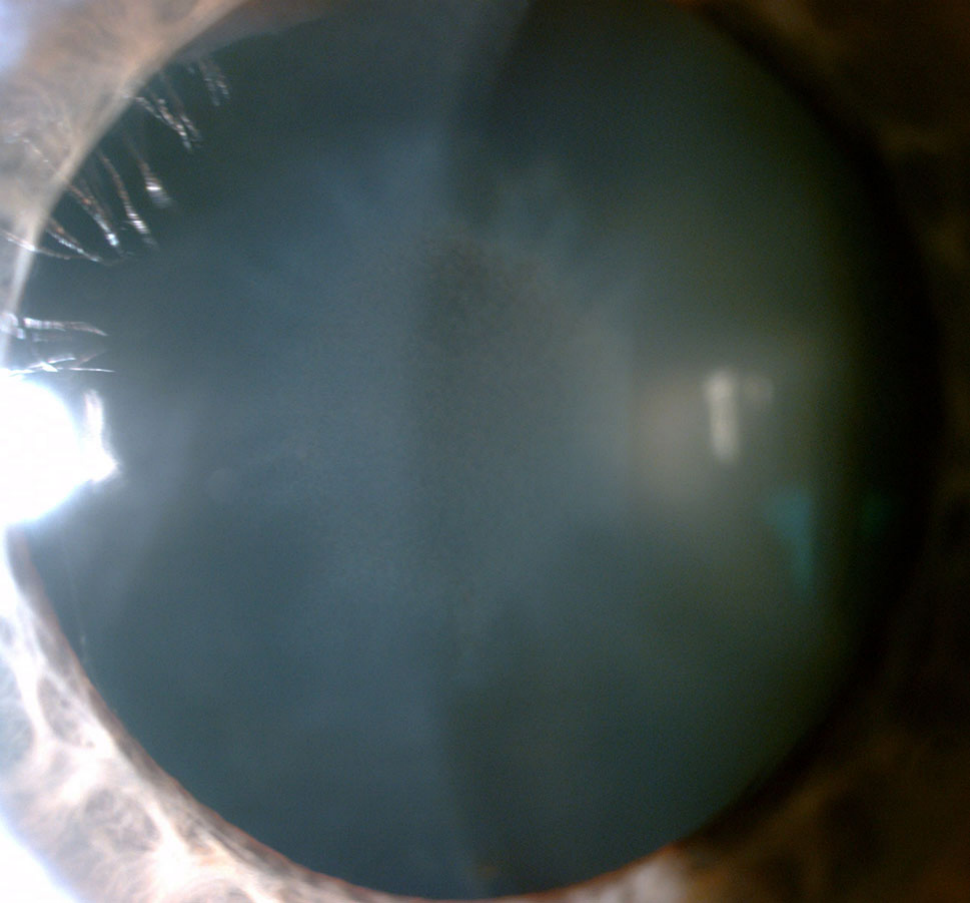
Sunflower cataract (SC) seen with slit lamp at time of WD diagnosis

Wilson’s disease (WD; OMIM No. 277900) is a genetic disorder of copper metabolism that results in copper accumulation in many tissues, mainly the liver, brain, cornea, and kidney. There is often secondary damage of affected organs and clinical manifestations related to the damaged organs [1–5]. The hepatic, neurological, and psychiatric signs and symptoms of WD are well described and characterized [1–7]. However, aside from the Kayser–Fleischer ring (K-F ring), which is produced by copper deposition in Descemet’s membrane and is regarded as a typical sign of WD, ophthalmologic signs of WD are rarely mentioned in WD research papers [1–5, 8–14]. Sunflower cataract (SC) is considered a second ophthalmic sign of WD and has been called pathognomonic for WD [8–14]. SC consists of a thin, centralized opacification that is located directly under the anterior capsule and encompasses between one-third and one-half of the anterior lens pole surface area. In all cases, the central opacification is surrounded by additional, secondary opacifications arranged in ray-like structures around it. This pattern resembles a sunflower, with a large central disk surrounded by petals (Fig. 1).

The K-F ring is an important clinical sign that is included in WD diagnostic algorithms and measures of treatment efficacy [1]. SC was initially described as a pathognomonic sign for WD almost 90 years ago [9], and this description has been repeated often in WD review articles [1–5]. However, SC is not included in the diagnostic criteria for WD and has been reported rarely, even in case reports [1, 14]. Potential reasons for the low occurrence of SC in WD patients are that it is an infrequent occurrence or that there is a lack of involvement of ophthalmologists who are experienced in SC diagnosis [9–13]. Because the ophthalmological manifestations of WD are reversible, the aim of our study was to analyze the occurrence of SC in a cohort of untreated, newly diagnosed WD patients in order to verify the importance and frequency of this sign in association with WD.

## Materials and methods

### Patients and methods

We examined 81 consecutive, newly diagnosed WD patients between January 2010 and May 2015. All patients were diagnosed with WD according to international criteria [1]. Presymptomatic and neurologically and hepatically predominant forms of the disease were distinguished according to the presence and intensity of clinical signs and symptoms at the time of diagnosis, as previously described [3]. The study was approved by the bioethics committee of the institution, and written informed consent was obtained from all patients.

The one ophthalmologist conducted all of the examinations. All patients underwent formal ophthalmological examinations, including best-corrected visual acuity (BCVA), near vision, and slit lamp examination. The patients’ pupils were then dilated (1 % tropicamide), and lens examination was performed. Special attention was paid to lens transparency, and the occurrence of SC and K-F ring was noted.

### Statistical analysis

All data were analyzed with Statistica v.10 (Stat Soft Inc., Tulsa, OK, USA). Mean, range, percentage, and standard deviation (SD) were measured for descriptive summary statistics.

## Results

Demographic and clinical data of the WD patients involved in the study are presented in Table 1. In our analysis of 81 WD patients, according to ophthalmological presentation of the disease, SC was found in only one WD patient (1.2 %), who exhibited an exclusive hepatic presentation. SC was found to have disappeared after 1 year of WD treatment with *d*-penicillamine.

**Table 1:** Demographic and clinical characteristics of the 81 analyzed WD patients

Gender	
Men (*n*)	34
Women (*n*)	47
Age at onset of WD symptoms (years)	28.6 ± 11.4
Age at WD diagnosis (years)	32.3 ± 11.8
Phenotypic forms of WD	
Neurological (*n*)	34
Hepatic (*n*)	33
Asymptomatic (*n*)	14
Baseline copper metabolism parameters	
Ceruloplasmin, mg/dl (normal range 25–45 mg/dl)	13.79 ± 6.1
Serum copper level, µg/dl (normal range 70–140 µg/dl)	60.23 ± 31.2
Urine copper excretion, µg/24 h (normal range 0–50 µg/24 h)	328.6 ± 854.9

The patient’s SC did not impact visual acuity. The BCVA of the affected WD patient was 0.9 in the right eye, which was the more affected lens, and 1.0 in the left eye, with full near vision acuity. SC disappeared from both eyes after treatment for WD, with both eyes reaching 1.0 BCVA upon examination. We did not detect any other disturbances in lens transparency.

K-F ring was identified in 67.9 % (55/81) of the examined patients, and its frequency depended upon the phenotypic presentation of the disease. It was detected in 100 % (34/34) and 57.5 % (19/33) of patients with neurological and hepatic manifestation of the disease, respectively. Only 14.2 % (2/14) of asymptomatic patients had K-F ring.

## Discussion

The ocular symptoms K-F ring and SC have historically been considered pathognomonic for WD [1]. The occurrence of K-F ring in WD is well documented, and it is a key sign in diagnostic algorithms for the disease [1]. SC, first described in 1922 by Siemerling and Oloff [9], was also initially postulated as a sign associated with WD [1]. However, apart from single case reports and two small ophthalmological studies (53 and 32 nonhomogenous WD patients), which presented conflicting results regarding SC occurrence (1.9–17 %), there have been no reports documenting the occurrence and significance of SC in WD [11–14].

To our knowledge, this was the first ophthalmological study in which the aim was to verify the occurrence and significance of SC in a relatively large cohort of newly diagnosed, untreated WD patients. We found a 1.2 % occurrence of SC in untreated WD patients. According to this finding, SC is a very rare and perhaps even incidental ophthalmological finding in WD that, as with other copper accumulation WD symptoms (e.g., K-F ring and WD-associated brain magnetic resonance imaging changes), may disappear during the course of disease treatment (as in our case) [1–3, 15, 16].

The presence of SC appeared to have a limited effect on patient visual acuity. This should be emphasized, as classical cataracts usually have a marked effect on visual acuity. Cataract is caused by irreversible protein changes in the lens, leading to diminishing lens transparency and, consequently, reduced visual acuity. SC is not a “true” cataract, as it is caused by reversible copper deposition under the anterior capsule of the lens [10–14, 18]. Furthermore, we found no cases of “true” cataract in our group; this is probably due to the young age of the WD patients in this population.

In any discussion of the significance and occurrence of SC as a medical symptom, it is worth mentioning that similar ocular symptoms may occur in eyes with foreign bodies, including copper [19–21]. High copper ion levels in intraocular foreign bodies (IOFBs) may lead to chalcosis bulbi. This phenomenon is dependent on the copper ion concentration in IOFBs. If the copper concentrations level is over 85 % it can produce inflammation with hypopyon, sterile endophthalmitis, and rapidly progress to phthisis. Chronic mild chalcosis (caused by IOFBs with copper concentration lower than 85 %) can produce K-F ring, SC and anterior chamber crystals [19–21]. However, unlike K-F ring, SC has not yet been reported in other medical conditions in which increased serum copper levels occur (e.g., neoplastic disorders and estrogen intake) [17, 22–24]. In patients with WD, the occurrence of SC has always been reported in patients with K-F ring, with both acting as ocular WD signs; to our knowledge, SC has never been reported as an isolated symptom in WD [17–24].

Our study also demonstrated the occurrence of K-F ring in WD patients; this is concordant with the results of previous studies. The frequency of K-F ring in WD patients is well documented and particularly affects patients with neurological presentation of the disease. However, our results also highlight the limitations of ophthalmological examinations when diagnosing WD in patients without neurological symptoms (especially presymptomatic patients) [1, 2].

A limitation of our study is that the group of analyzed patients could have been larger; however, this is the largest study group thus far of newly diagnosed WD patients who were examined for the presence of SC. Furthermore, our cohort was a homogenous group of patients who had not been treated previously.

## Conclusions

SC is a very rare symptom of WD that may disappear following treatment. This may be of interest to specialists involved in the diagnosis and treatment of WD, as it is a rare WD symptom that may sometimes aid in definitive diagnosis [25]. The presence of SC, in combination with K-F ring could point physicians toward a WD diagnosis. However, due to the extreme rarity of its occurrence, as well as its occurrence in other medical conditions, such as the presence of foreign intraocular bodies containing copper, it should not be considered a pathognomonic ocular sign of WD.
